# Assessing Health Literacy Among Hospitalized Patients: A Cross-Sectional Study in Silesia, Poland

**DOI:** 10.3390/healthcare13060593

**Published:** 2025-03-08

**Authors:** Kamila Jaroń, Mateusz Grajek, Joanna Kobza, Mateusz Rozmiarek

**Affiliations:** 1 Department of Public Health, Faculty of Public Health in Bytom, Medical University of Silesia in Katowice, 41-902 Bytom, Poland; kamila.jaron@gmail.com (K.J.); jkobza@sum.edu.pl (J.K.); 2 Department of Sports Tourism, Faculty of Physical Culture Sciences, Poznan University of Physical Education, 61-871 Poznan, Poland; rozmiarek@awf.poznan.pl

**Keywords:** health literacy, healthcare, communication, health promotion

## Abstract

**Background:** Health literacy includes the basic competencies needed to improve the quality of communication with medical personnel. **Objective:** The purpose of this study was to analyze the level of health literacy of hospitalized patients and to assess the impact of factors such as age, gender, education, place of residence, the ability to question the doctor about the information provided, and the evaluation of communication with medical personnel on their health literacy. **Material and methods:** The study was cross-sectional, and a specially designed questionnaire was used to collect data. Patients’ level of health literacy was assessed using the functional, communicative, and critical health literacy (FCCHL) scale. The study included 203 independent adult patients. **Results:** Higher levels of health literacy were observed more often in younger age groups, among women, people with higher education, and residents of large cities. Patients with a higher level of health literacy rated their communication with medical personnel higher, were more active in communicating with doctors, and asked questions more often, indicating that they were more involved in the medical decision-making process. A larger percentage of these patients believed that their consent to medical services was informed compared to patients with lower health literacy. In addition, patients with higher health literacy were more likely to express the belief that the medical staff spent enough time with them during their visit. **Conclusions:** The study’s findings underscore the importance of strengthening health education, which can help reduce health literacy disparities and improve health care quality.

## 1. Introduction

Health literacy, often referred to as health competence or functional health knowledge, is a multifaceted concept that encompasses an individual’s ability to understand, assess, and utilize health-related information effectively. It plays an essential role in making informed and accurate decisions regarding one’s health, particularly during a patient’s stay in a hospital or healthcare facility [1,2,3]. The World Health Organization (WHO) defines health competencies as cognitive and social skills that shape the motivation and ability of individuals to acquire, comprehend, and apply information that is necessary to maintain and enhance health. These competencies go far beyond the basic capacity to read, write, or understand health-related texts. They also encompass the ability to critically assess and evaluate the validity of health information, which is increasingly vital in today’s information-rich environment, where misinformation and confusing health messages are prevalent [4,5]. This critical evaluation ensures that individuals can discern between reliable and unreliable health advice, which is crucial in making decisions that can affect their health outcomes [5]. These skills are essential not only for accessing the right services but also for ensuring that individuals can participate actively in discussions about their health, leading to more personalized and effective care. An equally important facet of health literacy is the communication between patients and healthcare professionals. Encouraging healthcare providers to recognize and adapt to patients’ levels of health literacy can have a profound impact on the effectiveness of treatment, adherence to prescribed therapies, and overall patient satisfaction. When healthcare professionals understand the specific needs and capabilities of their patients in terms of health literacy, they are better positioned to tailor their communication strategies, making medical information more accessible and comprehensible. This fosters trust and collaboration, which are crucial for the successful delivery of care [3].

Effective communication is central to the healthcare process, influencing not only the practical aspects of medical treatment but also the psychological and emotional well-being of patients. Strong communication can improve therapeutic outcomes by ensuring that patients fully understand their diagnoses, treatment options, and the importance of adhering to medical advice. It also plays a crucial role in patient satisfaction, as patients who feel heard and understood are more likely to trust their healthcare providers and remain engaged in their treatment plans [6,7]. Therefore, health literacy is not just an individual attribute but also a shared responsibility between patients and healthcare providers, where mutual understanding and effective communication are essential components of high-quality care. As the literature indicates [3], health competence is not solely based on individuals’ skills and competencies but also depends on environmental factors, including people’s living environments.

Social determinants of health (SDoH), such as education, gender, economic status, and migration status, play a crucial role in shaping patients’ levels of health literacy. Given the strong connection between health literacy and social determinants of health (SDoH), it is essential to consider these factors when evaluating disparities in healthcare access, patient engagement during doctor–patient communication, and the process of providing informed consent. Patients with lower levels of health literacy, which are often influenced by factors such as advanced age, lower levels of education, or lower socioeconomic status, may face additional difficulties in understanding medical information and making informed decisions about their health. Addressing these disparities through tailored communication strategies and patient-centered approaches can enhance both the quality of interactions with medical professionals and the ability to provide truly informed consent, which is a key element of proper communication between doctor and patient [3,4,7].

The purpose of this study was to analyze the level of health literacy of hospitalized patients and to assess the influence of factors such as age, gender, education, place of residence, and the ability to elaborate and ask the doctor about the information provided and to evaluate the way they communicate with the medical staff on their health literacy. Patients’ level of health literacy significantly impacts the quality of doctor–patient communication. More specific research questions were posed to fulfill the purpose of the study:

1. Which social determinants of health influence patients’ health literacy levels?
2. Are there differences in perception and evaluation of communication with medical personnel among patients with lower and higher health literacy?
3. Are patients with higher health literacy more likely to actively participate in the treatment process than patients with lower health literacy?
4. How does patients’ level of health literacy impact their ability to make informed health decisions during hospitalization?

The results highlight the importance of a personalized approach to patients with lower health literacy, especially regarding access to medical information and improved communication. In addition, they provide a basis for further research into educational interventions and improving the quality of communication in healthcare, which, in the long term, may contribute to increasing the efficiency of the healthcare system.

## 2. Materials and Methods

### 2.1. Design of the Study

The present study employed a quantitative, cross-sectional design, with data collected through a specially designed questionnaire. The aim of the study was to evaluate the level of health literacy among hospitalized patients and examine the influence of social determinants of health, as well as communication with medical staff, on their health literacy.

### 2.2. Data Collection Procedures

Data were collected using a study-specific designed questionnaire, a random sample was selected, and the authors conducted the survey personally. The study was conducted between November 2021 and March 2022, with the peak of the fourth wave of the COVID-19 pandemic occurring in late November 2021. During this time, access to hospitals for external visitors was significantly restricted. Due to the unpredictable course of the pandemic, conducting surveys in hospital wards was not possible during certain periods. Consequently, the study was carried out in a single medical facility—a rehabilitation clinic in Silesia, Poland—using a face-to-face survey method. Patients from various hospital wards are referred to the clinic, which facilitated data collection and allowed for a more comprehensive assessment of patients’ health literacy.

### 2.3. Population

The study included 203 independent adult patients. The main criteria for inclusion in the study were being 18 years old or older and being in good health. Participation in the study was voluntary and anonymous, and the eligibility criterion included only independent people who did not require assistance with daily activities such as grooming or mobility. This selection made the group representative in the context of independent patients, although it excluded those requiring more intensive care. To check the sample’s representativeness, a minimum sample size was calculated for the hospitalized patient population during the period analyzed. With a 95% confidence level and an acceptable estimation error of 5%, the minimum sample size was 138 patients. In conclusion, a sample of 203 patients is sufficient to obtain reliable results in the context of this specific group, and the sample size is enough for the population of hospitalized patients.

Patients were categorized into two groups—those with lower and those with higher health literacy—which allowed for a detailed examination of how varying levels of health literacy influence the reception and processing of health information, as well as patients’ ability to actively engage in the treatment process. Patients were also divided into four groups according to their place of residence: rural area, small city (up to 20,000 residents), medium city (20,000–100,000 residents), and large city (over 100,000 residents). Further stratification of the patient population sample was carried out by age and by educational level.

### 2.4. Instrument

The survey form was pre-tested on a small group of patients to gather feedback on the clarity of the questions, the time required to complete the survey, and the relevance of the topics addressed. Respondents were informed about the study’s objectives and gave informed consent to participate. All those who qualified for the study agreed to complete the questionnaire.

The first demographic part of the questionnaire included questions on fundamental data, such as age, gender, and place of residence. The second part, related to individual factors, included questions about the level of education, the presence of chronic diseases, the hospital wards where the respondents were hospitalized, the length of their hospital stay, and the province where the hospitalization occurred. The last part consisted of 26 questions about patients’ communication with medical staff and relatives, their well-being during their stay in the hospital ward, and health literacy. The time allotted to complete the questionnaire was about 15 min.

The survey, in the category of closed-ended questions, used single-choice questions, and most questions used a Likert scale of five ranks (“definitely yes”, “rather yes”, “hard to say”, “rather no”, and “definitely no”). A point scale from 0 to 5 was used to assess the manner of communication between doctor and patient, and the respondents’ self-perception during hospitalization was assessed using the five-point well-being index developed by the World Health Organization (WHO-5). On the other hand, patients’ health literacy was assessed using the functional, communicative, and critical health literacy (FCCHL) scale. This scale is designed to measure a broad spectrum of health literacy. It considers three key skills: functional literacy and the basic reading and writing skills necessary to understand health information. At this level, the patient can read and understand the content of medication leaflets, forms, or messages provided by medical personnel [8]. Communicative literacy includes advanced skills in communicating and interacting with the health care system. The patient can make informed health-promoting decisions by actively interacting with medical professionals [8]. Critical literacy involves analyzing the information obtained to make optimal health decisions. Patients with this skill are capable of advanced health promotion and disease prevention activities [8]. The FCCHL scale was initially developed to assess health literacy in patients with type 2 diabetes [9] and has over time been validated in various populations, including citizens of France [10], The Netherlands [11], Germany [12], and Poland [8]. The FCCHL scale represents the full range of health literacy.

The response options were modified by the authors from a 4-point scale assessing the frequency of a given situation (“never” to “often”) to a 5-point scale indicating the respondent’s degree of agreement with the item, where one meant “strongly disagree” and five meant “strongly agree” [13]. The modification of the response options from a 4-point scale to a 5-point scale has also been applied in other studies. Previous research has utilized and validated the 5-point version of the FCCHL scale, demonstrating its reliability and effectiveness in assessing health literacy [13,14,15,16,17]. The 5-point scale allows for a more detailed capture of differences in health literacy levels, which may enhance the accuracy of the results. This scale also provides respondents with an option for neutrality, enabling them to express an ambivalent opinion instead of forcing a choice between a positive or negative response. Additionally, it is more intuitive and easier for participants to understand.

For all 14 questions, the mean health literacy score was calculated as the average of the subscale scores [8]. Patients were divided into two groups based on the median FCCHL scale score. Higher scores indicated higher levels of health literacy, except questions from the functional subscale of health literacy, where the lowest scores indicated higher levels of health literacy. The functional subscale values were reversed to standardize the interpretation of the results. Therefore, patients whose FCCHL score was below the median were classified as having a lower level of health literacy, and those whose score was equal to or above the median were classified as having a higher level of health literacy. No clear consensus has been reached in the literature regarding the division of those with lower or higher levels of health literacy. According to some researchers, a lower level of health literacy has been defined as a score below the median, while other researchers have considered a value of 3 as the cutoff point for a low or high level of health literacy [11,16,18,19].

### 2.5. Statistical Analysis

The obtained results of the study were subjected to statistical analysis. The overall assessment of how the subjects communicated with the medical staff was determined as the average of the scores of the tested aspects of communication (rated by the subjects on a scale of 0–6). The normality of the distribution of the FCCHL subscale scores was assessed using the Shapiro–Wilk test. Differences between health literacy subscales (functional, communication, and critical) were evaluated using Friedman’s ANOVA analysis of variance and Wilcoxon’s paired rank order test with Bonferroni correction. The division of subjects into those with lower and higher levels of health literacy was based on the median FCCHL score (subjects whose FCCHL score was below the median were classified as those with lower levels of health literacy). The relationship between the level of health literacy and selected variables was evaluated using the chi-squared test and Yates’s chi-squared test. A significance level of *p* < 0.05 was adopted to indicate the existence of statistically significant differences or relationships. Database and statistical tests were performed using Statistica 9.1 (StatSoft, Krakow, Poland) and PQStat 1.8.2 computer software.

To evaluate the internal consistency of the questionnaire, a Cronbach’s alpha test was conducted for the section focused on health literacy. The Cronbach’s alpha value was found to be 0.84, indicating strong reliability of the instrument. This value falls within the acceptable range for social research, where a score of 0.7 or above is generally deemed adequate to confirm consistency among the questions. This suggests that the items in the questionnaire were consistent with one another and effectively measured the same construct, which in this case was health literacy.

### 2.6. Ethical Considerations

The study was conducted by the provisions of the Declaration of Helsinki, and its implementation did not require the approval of the Bioethics Committee of the Silesian Medical University in Katowice, which was confirmed by Decision No. PCN/CBN/0052/KB/187/22; 12 July 2022.

The participants were provided with detailed information about the study’s objectives, its procedure, and potential risks associated with participation. They were also informed that participation was entirely voluntary and that they could withdraw at any time without any consequences. No personal data, such as names, addresses, or contact information, were collected during the study, ensuring that no personal information could be linked to any specific participant. The surveys collected during the study are stored securely.

## 3. Results

The surveyed patients included 132 women (65.0%) and 71 men (35.0%), with a high school education making up the largest group at 44.4% (N = 90). Those with primary education accounted for 3.0% (N = 6), with vocational education 25.6% (N = 52), and with higher education 27.0% (N = 55). In terms of place of residence, the most significant number of patients came from medium-sized cities (20–100 thousand residents) 53.7% (N = 109). This was followed by residents of large cities (>100 thousand residents) 20.2% (N = 41), small cities 19.2% (N = 39), and rural areas 6.9% (N = 14). Among the respondents, 24.6% (N = 50) were up to 45 years old, 26.6% (N = 54) were between 46 and 55 years old, 20.2% (N = 41) were in the 56–65 age group, and 28.6% (N = 58) were over 66 years old (Table 1).

Patients included in the survey were hospitalized mainly in the Silesian province (98.0%; N = 199), while 2.0% (N = 4) of the respondents were in hospitals located in the Opolskie, Malopolskie, Podkarpackie, and Lower Silesian provinces. The distribution of patients by hospital department was as follows: 18.0% (N = 36) of people were treated in the orthopedic ward, 14.0% (N = 29) in the gynecology ward, 14.0% (N = 29) in the cardiology ward, 11.0% (N = 23) in the neurology ward, 9.0% (N = 18) in the general surgery ward, 7.0% (N = 14) on the rehabilitation ward, 6.0% (N = 13) on the internal medicine ward, 6.0% (N = 12) on the urology ward, 3.0% (N = 7) on the rheumatology ward, and 3.0% (N = 7) on the pulmonology ward. Single hospitalizations were in other departments, such as dermatology, ENT, ophthalmology, nephrology, diabetology, endocrinology, psychiatry, oncology, and pregnancy pathology.

The majority of hospitalized patients were informed about their condition (89.2%; N = 181), as well as about proposed and possible diagnostic methods and the anticipated consequences of their use or omission (87.2%; N = 177). Similarly, a high percentage of people received information about the proposed and possible treatment methods and the anticipated consequences of their use or omission (88.7%; N = 180) about the results of treatment and prognosis (88.2%; N = 179) (Table 2).

The vast majority of respondents (55.2%; N = 112) declared that they would rather have had the opportunity to clarify to ask the doctor about the information communicated, 33.0% (N = 67) of people definitely had such an opportunity, and 2.0% (N = 4) of respondents said that they would rather not have such an opportunity. In comparison, 9.8% (N = 20) could not answer this question (Table 2).

Most respondents (92.6%; N =188) indicated that consent for the medical services provided was informed, meaning that it was preceded by comprehensive information. A neutral stance on this issue was taken by 6.4% (N = 13) of people, while 1.0% (N = 2) of respondents expressed the opposite opinion (Table 2).

In addition, 81.0% (N = 165) of all respondents indicated that the doctors took enough time for the examination. However, 6.0% (N = 11) of all respondents believed otherwise, and 13.0% (N = 27) of respondents had no opinion on the subject (Table 2).

After classifying the patients into those with higher and lower levels of health literacy, the relationship of the levels so determined with the independent variables, including age, gender, education, and place of residence, was checked. The analysis showed a statistically significant relationship between the level of health literacy and the age, gender, education, and place of residence of the respondents. Among the respondents under 45 years old, a significantly higher percentage of people (78.0%; N = 39) had higher health literacy compared to older people. On the other hand, in the group of respondents over 66 years old, the highest percentage of people with lower levels of health literacy was observed (74.1%; N = 43). In terms of gender, women were more likely to have higher health literacy (58.3%; N = 77) than men (35.2%; N = 25). Education also played a significant role, with the highest number of respondents with higher health literacy reported among those with a higher degree (83.6%; N = 46), while the highest number of respondents with lower health literacy was reported among those with primary and vocational education (86.2%; N = 50). The place of residence was also significantly correlated. Most respondents living in large cities (more than 100,000 residents) had higher health literacy (78.1%; N = 32), while the most significant percentage of respondents living in small towns (up to 20,000 residents) had lower health literacy (82.1%; N = 32) (Table 3).

Moreover, the survey results indicate that those with higher health literacy were more likely to have the opportunity to elaborate to ask the doctor about the information provided (95.1%; N = 97), which indicates that they were more active in communicating with medical personnel, compared to those with lower health literacy (81.2%; N = 82). Analysis of the results also showed that patients with higher health literacy were more likely to rate their consent for a specific medical service as informed, which indicated that it was preceded by the provision of comprehensive information (99.0%; N = 101). Fewer respondents with lower health literacy shared this view (86.1%; N = 87). Respondents characterized by higher health literacy were also more likely to express the belief that the medical staff spent enough time with them during the examination (87.2%; N = 89) compared to patients with lower health literacy (75.2%; N = 76) (Table 4).

Respondents also rated their communication with doctors on a 6-point scale, from very bad to very good, including doctors’ professionalism, trustworthiness, simple and understandable language, answering questions, openness, friendliness, providing sufficient emotional support, and adherence to professional confidentiality (Table 5).

The overall evaluation of the manner of communication was calculated as the average of the sub-ratings and then the respondents were divided according to a cut-off point of four. The results show that a higher percentage of those with higher health literacy (81.4%; N = 83) rated communication with medical personnel above four, compared to those with lower health literacy (52.5%; N = 53) (Table 6).

Analysis of the results of patients with higher and lower health literacy, based on the overall FCCHL score, which is the average of the three subscales, showed statistically significant differences between patients’ levels of functional, communicative, and critical health literacy (*p* < 0.001). The result of the analysis indicates that the level of functional health literacy is statistically significantly lower than the level of communicative health literacy but, at the same time, higher than the level of critical health literacy. In turn, the level of communicative health literacy was significantly higher than both functional and critical health literacy levels. As a result, the analysis reveals that the level of critical health literacy is significantly the lowest among the three levels of health competence studied. In contrast, the level of communicative health literacy significantly exceeds the results in the other two subscales (Table 7).

## 4. Discussion

Health literacy is a crucial factor influencing variations in patients’ knowledge levels, self-care abilities, effectiveness in adhering to medical advice, and participation in health-promoting behaviors [20]. Individuals with higher health literacy can plan lifestyle changes and make decisions based on various information sources. Despite the crucial importance of health literacy to quality of life, many countries still struggle to raise it among patients. In Canada, as many as 88% of older adults and 60% of adults lack health literacy, according to a study designed to review the scientific literature, negatively affecting their ability to make informed decisions and exercise control over their health. Similar challenges apply to high-income countries in Europe. The level of health literacy in European societies varies widely. Research emphasizes the need to implement effective educational strategies and activities that support the development of health literacy among different social groups [20].

Lower levels of health literacy are often associated with less use of preventive services and more reliance on emergency care. This pattern of behavior increases the risk of poorer health outcomes [20]. It can also be associated with low socioeconomic status, unemployment, lack of education, or severe consequences such as higher mortality, higher risk of depression, and lower adherence to medical recommendations [21]. Higher levels of health literacy, in turn, promote more informed health decisions and increase patient involvement in the healthcare process [21].

The authors’ study indicates that health literacy is associated with social determinants of health, particularly higher education and living in larger cities. The highest percentage of individuals with higher health literacy was found among patients with higher education and those residing in large cities. These results are consistent with an analysis of the literature, which indicates that low education is a significant determinant of lower health literacy. A study conducted in the United States that aimed to use the Extended Gastwirth Index to quantify differences in health literacy using population characteristics indicative of health disparities (education, income, age, race, and gender) indicated that income and education contribute to overall differences in health literacy [22]. People with higher education are more likely to enjoy good health than those with lower education. A study conducted in the Netherlands using the Dutch Health Literacy Questionnaire, involving a nationwide sample of people with chronic somatic diseases and physical disabilities, found that higher levels of education correlate with higher health literacy scores. This includes better assessment of health information and the ability to navigate the health care system [23]. Another cross-sectional study, using an online questionnaire for the FCCHL survey and aimed at assessing the reliability and accuracy of the French translation of the FCCHL scale, found that those with lower education had lower levels of health literacy compared to those with higher education [10].

Regarding place of residence, the literature does not provide a clear answer as to whether those living in large cities have a higher level of health literacy. On the one hand, residents of rural, small towns may have limited access to health information sources and use them less frequently than residents of larger cities. Numerous barriers, such as a lack of specialist doctors and limited exposure to the media, make it difficult for rural residents to access relevant health information, especially for those with lower levels of health literacy [24]. On the other hand, a study using questionnaires assessing various aspects of health literacy in Germany found higher levels of health literacy in rural regions, suggesting that rural residents might possess higher health literacy as well [25]. Although health literacy levels cannot be determined solely by region, environmental factors play an essential role.

The authors’ study findings suggest that health literacy may shape patients’ perceptions of whether they received sufficient time and attention during their medical visits. Patients with higher health literacy may feel more confident during medical conversations and in interacting with healthcare providers, which may reinforce their belief that they receive sufficient attention during visits. In contrast, individuals with lower health literacy may struggle to effectively communicate their concerns, potentially leading to the impression that they are not given enough time. This highlights the importance of ensuring that all patients feel adequately supported during medical interactions. Longer consultations may be associated with better quality of care and more favorable health outcomes, especially among those with lower levels of health literacy. Studies in England have found no association between consultation length and patients’ communication experiences. This suggests that the length of the visit itself does not necessarily reflect the quality of the visit. Nevertheless, physicians should individually adjust the amount of time spent with patients during the visit, especially those with low levels of health competence. This approach can help the patient better understand the information provided by the medical staff. In addition, further research is needed to better understand the potential benefits of longer consultations, especially in the context of differences related to patients’ level of health literacy [26].

Little is known about the relationship between health literacy and questioning behavior during medical visits. The study findings suggest that patients with higher health literacy tend to take a more active role in their interactions with healthcare providers. They are more likely to ask questions, seek clarification, and participate in the exchange of medical information. This increased engagement reflects a stronger doctor–patient relationship and a more informed approach to health management and decision-making. In a cross-sectional observational study conducted to record patient visits to orthopedic hand surgeons in the northeastern United States, patients’ level of health literacy was assessed using the Newest Vital Sign (NVS) tool. The study results showed that patients with limited health literacy asked fewer questions about their medical care than those with adequate health literacy. Limited health literacy has been identified as a barrier to effective patient engagement in the surgical care process. These implications are significant because asking more questions can provide patients with more information, and the difference in the number of questions observed in the study between patients with limited and adequate health literacy revealed a potential source of inequity in access to health information. In addition, the study indicates that people with limited health literacy often hide the problem from their families or avoid seeking medical care because of embarrassment. Simple interventions, such as actively encouraging patients to ask questions, can significantly improve the quality of patient–doctor communication, thereby increasing access to information and supporting better health decisions [27].

Another study based on a literature analysis highlights the relationship between the level of health literacy and the quality of interactions with medical personnel. Health literacy was considered a key element in the communication process between patient and doctor. The article emphasizes that an adequate level of health literacy enables patients to understand better the information provided, which improves health decision-making [28]. A study conducted by the authors showed that patients with higher health literacy rated the quality of their communication with the doctor higher and were more likely to consider their consent for a specific medical service as informed. This means that the medical staff provided them with comprehensive and understandable information, underscoring the importance of effective communication in the doctor–patient relationship, especially in the context of differences in health literacy.

Since the study focuses on independent individuals and non-independent individuals, patients with disabilities may face specific challenges related to health communication and decision-making; the generalizability of its findings may be limited. To overcome this limitation, future research should include a more diverse patient population. Such an approach could lead to a more comprehensive understanding of differences in health literacy and contribute to the development of personalized and effective health interventions. At the same time, the study’s findings indicate that healthcare professionals should consider social determinants of health in the communication process and adapt their methods of conveying information to patients’ levels of health literacy. For individuals with lower health awareness, using simple language, incorporating visual aids, and clearly explaining key concepts can significantly enhance understanding and support effective communication.

In the context of improving communication between patients and healthcare professionals, as well as educational interventions aimed at increasing patients’ health literacy, there are several effective strategies and approaches that deserve special attention.

One important aspect is ensuring that healthcare professionals receive appropriate training, with a particular focus on developing communication skills and effective collaboration with patients. The training should include active listening, the ability to adjust communication style to the patient’s level of education, age, and individual needs, as well as providing clear explanations of complex medical terms and creating space for patients to ask questions. Such an approach significantly enhances the quality of interactions, promoting better understanding and greater patient engagement in the treatment process [29]. Moreover, educational programs for patients, such as health-related training and workshops, are an essential tool to help patients better understand their health condition and available treatment options and make informed decisions. Examples include programs focused on chronic diseases such as diabetes, hypertension, or heart disease, which help patients better manage their own health. Research shows that patients who have access to reliable information about treatment and lifestyle manage chronic conditions more effectively [30]. Additionally, technologies, including mobile applications and online platforms, can provide valuable support in the process of health education. Through these apps, patients can monitor their health, receive medication reminders, access educational materials, and obtain health advice. Proper use of technology can contribute to improving patients’ health literacy and enable them to make more informed health decisions. Examples of such apps include tools for tracking symptoms, test results, or physical activity levels, which engage patients in the treatment process [31]. Through education, effective communication, and access to modern technologies, patients can gain the tools they need to make more informed, well-considered decisions, which positively impacts their health and quality of life.

## 5. Strengths and Limitations

The conducted study should be considered innovative, as it addressed patients’ health literacy, a topic rarely analyzed in the literature, especially using the FCCHL scale in Poland. The limited number of available studies made comparing the results of previous analyses difficult. The study included only independent patients, which limited the ability to generalize the results to other groups of patients, such as those with less autonomy in making health decisions. Another limitation was the time between hospitalization and respondents’ survey completion, which may have affected their ability to recall detailed interactions with medical personnel. Moreover, the survey was conducted at a single facility, a rehabilitation clinic, which limited our ability to consider the diverse communication dynamics present in other medical settings, such as emergency or intensive care units, where working conditions and patient circumstances may differently affect hospitalized patients with varying levels of health literacy. To increase the generalizability of the findings, further research is needed that includes a variety of medical settings. Future studies should be multicenter and include more diverse and dynamic settings, such as emergency or intensive care units. Additionally, using mixed methods that combine quantitative research, such as surveys, with qualitative research, like in-depth interviews or focus groups, could provide richer data and better clarify the factors that differentiate patients’ health literacy levels.

Undoubtedly, the assessment of health literacy can be measured by various tests. One test is the Rapid Estimate of Adult Literacy in Medicine (REALM), and another widely used instrument is the Test of Functional Health Literacy in Adults (TOFHLA). Still, these instruments mainly focus on functional health literacy. In contrast, other instruments measure multidimensional health literacy, including the Health Literacy Questionnaire (HLQ) and the Functional Communicative Critical Health Literacy (FCCHL), which was selected by the authors for this study [12]. The FCCHL, originally developed for patients with chronic diseases, is also used to assess health literacy in hospitalized patients. A tool used to assess functional, communicative, and critical health aspects, it is particularly useful when patients need to quickly assimilate a large amount of information. The simple design and short questions make the tool suitable for those who may experience fatigue or stress during hospitalization.

## 6. Practical Implications

The survey points to the need for training for doctors and nurses in effective communication with patients, particularly in active listening skills and answering patients’ questions in a clear and accessible manner. The study results can be the basis for creating educational programs that make patients more active in communicating with their doctor. This can translate into greater involvement in the treatment process and better health outcomes. The study’s findings can also be incorporated into education and prevention campaigns emphasizing the importance of developing health literacy as a key component of adequate health care, especially in crises like epidemics. Implementing special programs, including support groups, one-on-one consultations, or access to health education materials, could improve understanding and applying medical information. Raising health literacy and actively involving patients with limited health literacy in decision-making should also become a significant goal for many health systems. Such a practice promotes a better understanding of medical information and builds trust between the patient and medical personnel.

The development and promotion of digital tools that support patients with various levels of health literacy also is recommended. Mobile apps and websites offering interactive content can provide personalized health information and assist in decision-making processes, allowing patients easier access to knowledge and resources. Moreover, governments and healthcare institutions should introduce policies that require the inclusion of health literacy strategies in public health campaigns and patient education programs. These actions may include preparing materials in simple language that are easy to read, as well as using multimedia tools that effectively reach a wide audience. Healthcare institutions should also verify written materials, ensuring readability, and adapt informed consent documents to patients’ comprehension levels. For patients with lower health literacy, as well as those who encounter difficulties navigating the healthcare system, involving caregivers and family members in the communication process is essential. Their support can significantly improve understanding of information and help in making more informed health decisions.

## 7. Conclusions

Analysis of the survey results showed a significant relationship between patients’ level of health literacy and demographic variables such as age, gender, education, and place of residence. Higher levels of health literacy were observed more often among younger age groups, women, people with higher education, and residents of large cities. These correlations indicate the need for an individualized approach to communicating health information by medical personnel, considering the diverse needs of different patient groups. In the communication process, doctors should primarily focus on providing information support to the elderly, patients with lower education, and residents of rural areas and small towns. Such an approach can help improve their health literacy, bridging the gap in access to information and the quality of health care provided.

Findings from the study indicate that patients with higher health literacy are more involved in the treatment process and better evaluate the quality of communication with medical personnel, which may lead to increased confidence in the services provided. Informed consent for treatment, more frequent questioning, and clarification of information suggest that higher levels of health literacy enable patients to take a more active role in medical decision-making, leading to better health outcomes. At the same time, the study underscores the need to increase health education efforts, especially among those with lower health literacy. Adapting the content and methods of communication to the needs of different patient groups can also help improve patients’ understanding of the treatment process, increase their satisfaction with medical care, and improve their effectiveness in communicating with medical personnel. It is also worth considering the need to support doctors in developing communication skills to more effectively identify the information needs of patients with different levels of health literacy and provide them with appropriate care during visits.

## Figures and Tables

**Table 1:** Characteristics of the studied group of patients.

		N	%
Age	M ± SD	55.5 ± 13.7	
	Me [Q1–Q3].	55 [46–68]	
	Min–Max	19–87	
	up to 45 years	50	24.6
	46–55 years	54	26.6
	56–65 years	41	20.2
	66 years and over	58	28.6
Gender	women	132	65.0
	men	71	35.0
Education	primary	6	3.0
	vocational	52	25.6
	secondary	90	44.4
	higher	55	27.0
Place of residence	village	14	6.9
	small city of up to 20,000 residents.	39	19.2
	medium city of 20–100 thousand residents.	109	53.7
	large city over 100,000 residents.	41	20.2
Total		203	100.00

**Table 2:** Patient information provided by medical staff and time spent during examination.

Obtaining Information from Medical Staff and Time Spent During Examination (Total Score)			
		N	%
Were you, upon admission to the hospital, informed of:	health	181	89.2
	proposed and possible diagnostic methods and foreseeable consequences of using/not using diagnostic methods	177	87.2
	proposed and possible treatment methods and foreseeable consequences of using/not using treatment methods	180	88.7
	treatment results/prognosis	179	88.2
After receiving the above information, did you have the opportunity to elaborate, to ask the doctor more about the information provided?	definitely yes	67	33.0
	likely yes	112	55.2
	hard to say	20	9.8
	rather not	4	2.0
	definitely not	0	0.0
Was your consent to a specific medical service informed, that is, preceded by the provision of comprehensive information?	definitely yes	102	50.2
	likely yes	86	42.4
	hard to say	13	6.4
	rather not	2	1.0
	definitely not	0	0.0
Do you feel that the medical staff spent enough time with you during the examination?	definitely yes	57	28.0
	likely yes	108	53.0
	hard to say	27	13.0
	rather not	10	5.0
	definitely not	1	1.0
Total		203	100.00

**Table 3:** Relationship between the level of health literacy (total score) and the independent variables analyzed.

Analyzed Variable			Health Literacy (Total Score)		Chi^2^ *p*
			Lower	Higher	
Age	(I) up to 45 years old	N	11	39	Chi^2^ = 29.291 *p* < 0.001 V = 0.380
		%	22.0%	78.0%	
	(II) 46–55 years old	N	26	28	
		%	48.1%	51.9%	
	(III) 56–65 years old	N	21	20	
		%	51.2%	48.8%	
	(IV) 66 years and over	N	43	15	
		%	74.1%	25.9%	
Gender	women	N	55	77	Chi^2^ = 9.873 *p* = 0.002 V = 0.221
		%	41.7%	58.3%	
	men	N	46	25	
		%	64.8%	35.2%	
Education	(I) primary/vocational	N	50	8	Chi^2^ = 55.701 *p* < 0.001 V = 0.524
		%	86.2%	13.8%	
	(II) secondary	N	42	48	
		%	46.7%	53.3%	
	(III) higher	N	9	46	
		%	16.4%	83.6%	
Place of residence	(I) village	N	9	5	Chi^2^ = 30.516 *p* < 0.001 V = 0.388
		%	64.3%	35.7%	
	(II) small city	N	32	7	
		%	82.1%	17.9%	
	(III) medium city	N	51	58	
		%	46.8%	53.2%	
	(IV) large city	N	9	32	
		%	21.9%	78.1%	

Note: V—Cramer’s V correlation is used to measure the association between two attributes.

**Table 4:** Patient engagement, informed consent, and time spent during examination, with a division of patients with lower and higher health literacy.

Analyzed Variable			Activity in Communication		Total	Chi^2^ *p*
			Definitely Yes/Rather Yes	Hard to Say/Rather Not		
Health literacy (total score)	lower	N	82	19	101	Chi^2^_Yates_ = 8.132 *p* = 0.004 V = 0.215
		%	81.2%	18.8%		
	higher	N	97	5	102	
		%	95.1%	4.9%		
			Informed consent			
			definitely yes/rather yes	hard to say/rather not		
Health literacy (total score)	lower	N	87	14	101	Chi^2^_Yatesa_ = 10.494 *p* = 0.001 V = 0.246
		%	86.1%	13.9%		
	higher	N	101	1	102	
		%	99.0%	1%		
			The amount of time spent on the patient’s examination			
			definitely yes/rather yes	hard to say/rather not		
Health literacy (total score)	lower	N	76	25	101	Chi^2^ = 4.052 *p* = 0.044 V = 0.154
		%	75.2%	24.8%		
	higher	N	89	13	102	
		%	87.2%	12.8%		

**Table 5:** Distribution of evaluations of the manner of communication with medical staff.

How to Communicate with Medical Staff	Evaluation											
	0 Very Bad		1		2		3		4		5 Very Good	
	N	%	N	%	N	%	N	%	N	%	N	%
He was reliable, professional	0	0.0	1	0.5	8	3.9	12	5.9	46	22.7	136	67.0
It was understandable and benefited from the simplicity of the message (plain language)	0	0.0	1	0.5	8	3.9	16	7.9	44	21.7	134	66.0
He answered the questions asked	0	0.0	1	0.5	7	3.5	18	8.9	42	20.6	135	66.5
He was characterized by openness and kindness	1	0.5	1	0.5	13	6.4	17	8.4	42	20.6	129	63.6
He kept professional secrets	1	0.5	1	0.5	5	2.5	11	5.4	39	19.2	146	71.9
Provided sufficient emotional support	2	1.0	3	1.5	12	5.9	21	10.3	38	18.7	127	62.6

**Table 6:** Overall evaluation of communication with medical staff, with a division into patients with lower and higher health literacy.

			Overall Assessment of How to Communicate with Medical Staff			
			Lower (Up to 4 Points)	Higher (Above 4 Points)		
Health literacy (total score)	lower	N	48	53	101	Chi^2^ = 19.165 *p* < 0.001 V = 0.307
		%	47.5%	52.5%		
	higher	N	19	83	102	
		%	18.6%	81.4%		

**Table 7:** Results of the analysis of patients’ health literacy (FCCHL) by functional, communication, and critical health literacy.

FCCHL	M	Me	Min	Max	Q1	Q3	SD	
Overall result	3.47	3.43	1	5	3.1	4.0	0.80	
(f)unctional health literacy	3.50	4	1	5	2.8	4.0	1.04	Chi^2^_ANOVA_ = 71.122 *p* < 0.001 f < co, f > cr, co > cr
(Co)communication literacy	3.80	4	1	5	3.6	4.0	0.76	
(cr)ytic health literacy	3.12	3	1	5	2.0	4.0	1.03	

M—mean; Me—median; Min—minimum; Max—maximum; Q1—lower quartile; Q3—upper quartile; SD—standard deviation; Chi^2^_ANOVA_—Friedman’s ANOVA; *p*—*p*-value.

## Data Availability

Data are contained within the article. Further inquiries can be directed at the corresponding author.
